# Consider Hereditary Angioedema in the Differential Diagnosis for Unexplained Recurring Abdominal Pain

**DOI:** 10.1097/MCG.0000000000001744

**Published:** 2022-08-15

**Authors:** Kyle Staller, Anthony Lembo, Aleena Banerji, Jonathan A. Bernstein, Eric D. Shah, Marc A. Riedl

**Affiliations:** * Massachusetts General Hospital; † Beth Israel Deaconess Medical Center, Harvard Medical School, Boston, MA; ‡ University of Cincinnati College of Medicine and Bernstein Allergy Group, Cincinnati, OH; § Section of Gastroenterology and Hepatology, Lebanon, NH; ∥ US HAEA Angioedema Center, University of California San Diego, San Diego, CA

**Keywords:** hereditary angioedema, unexplained abdominal pain, differential diagnosis

## Abstract

Health care providers are likely to encounter patients with recurrent unexplained abdominal pain. Because hereditary angioedema (HAE) is a rare disease, it may not be part of the differential diagnosis, especially for patients who do not have concurrent skin swelling in addition to abdominal symptoms. Abdominal pain is very common in patients with HAE, occurring in up to 93% of patients, with recurrent abdominal pain reported in up to 80% of patients. In 49% of HAE attacks with abdominal symptoms, isolated abdominal pain was the only symptom. Other abdominal symptoms that commonly present in patients with HAE include distension, cramping, nausea, vomiting, and diarrhea. The average time from onset of symptoms to diagnosis is 6 to 23 years. Under-recognition of HAE in patients presenting with predominant gastrointestinal symptoms is a key factor contributing to the delay in diagnosis, increasing the likelihood of unnecessary or exploratory surgeries or procedures and the potential risk of related complications. HAE should be considered in the differential diagnosis for patients with unexplained abdominal pain, nausea, vomiting, and/or diarrhea who have complete resolution of symptoms between episodes. As highly effective targeted therapies for HAE exist, recognition and diagnosis of HAE in patients presenting with isolated abdominal pain may significantly improve morbidity and mortality for these individuals.

Hereditary angioedema (HAE) is a rare, autosomal dominant disease characterized by unpredictable, recurrent swelling affecting the skin and submucosal tissue, including the gastrointestinal (GI) tract.^1^–^3^ Most cases of HAE are caused by mutations in the gene *SERPING1*, which encodes C1 inhibitor (C1-INH).^1^ This type of HAE is known as HAE with C1-INH deficiency or HAE-C1-INH. HAE-C1-INH can be divided into 2 subtypes on the basis of low levels of functioning C1-INH (type 1) and normal to high levels of nonfunctioning C1-INH and diminished C1-INH activity (type 2).^3^ The clinical presentation in the 2 HAE subtypes is heterogeneous, with no apparent phenotypic differences between subtypes and no clear correlation of symptom frequency or severity with the degree of C1-INH deficiency.^4^ HAE-C1-INH globally affects ~1:67,000 people,^3^,^5^,^6^ and symptoms typically start in childhood (mean age at onset, 8 to 12 y).^3^ C1-INH regulates factor XIIa and plasma kallikrein in the contact activation system of the coagulation pathway (Fig. 1).^7^ Plasma kallikrein cleaves high-molecular-weight kininogen, which results in the generation of bradykinin.^1^ Bradykinin subsequently binds to the bradykinin B2 receptor and increases vascular permeability, resulting in the swelling that occurs in patients with HAE-C1-INH. For simplicity, we will use HAE in this article to mean HAE-C1-INH.

**FIGURE 1:**
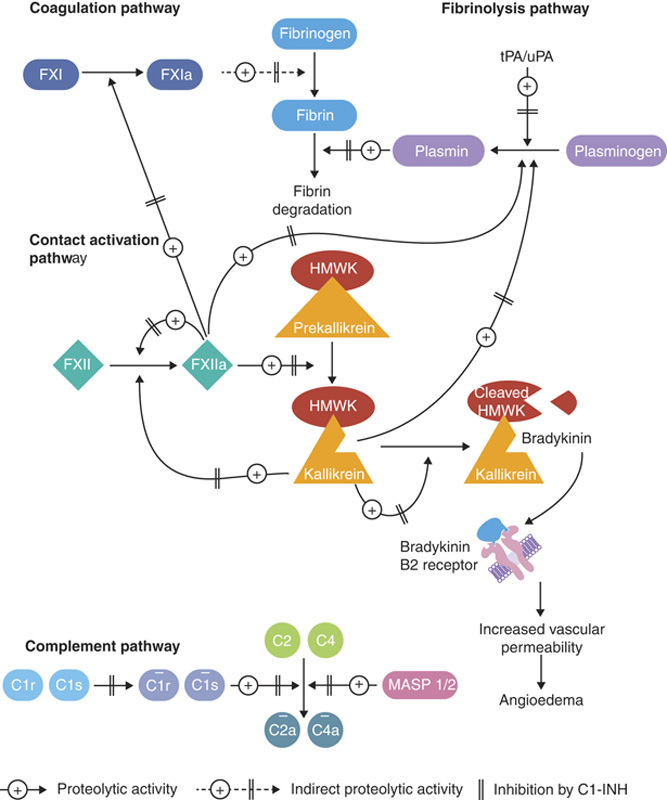
The lack of functional C1-INH affects the complement, contact, coagulation, and fibrinolytic pathways. Horizontal bars over the complement components indicate activation. Double vertical bars indicate inhibition by C1-INH. C1-INH indicates C1 esterase inhibitor; FXI, factor XI; FXIa, activated factor XI; FXII, factor XII; FXIIa, activated factor XII; HMWK, high-molecular-weight kininogen; MASP, mannose-binding lectin-associated serine protease; tPA/uPA, tissue/urokinase plasminogen activator. Reproduced with permission from Bork K. 7

HAE management guidelines developed by the US HAE Association Medical Advisory Board acknowledge that the management of HAE depends on health care providers recognizing possible symptoms of HAE, including abdominal symptoms from GI angioedema attacks.^8^ Guidelines for the diagnosis of HAE also have been published for Canada, Brazil, the United States, and internationally,^6^,^8^–^12^ but none of these specifically focus on the differential diagnosis of HAE with predominant GI symptoms.

Owing to the wide heterogeneity of clinical signs and symptoms of HAE as well as the variable frequency and severity of attacks,^13^ clinicians may be unfamiliar with the disease and may not include HAE in the differential diagnosis, meaning a diagnosis of HAE may be delayed for many years. Patients with HAE presenting with GI symptoms alone, without typical skin swelling, can lead to the diagnosis being overlooked. Undiagnosed patients with HAE are at a substantially higher risk of mortality from their disease.^14^ Mortality due to asphyxiation caused by laryngeal swelling was 29.4% in patients diagnosed with HAE (n=63/214) postmortem versus 3.3% for patients with a known diagnosis (n=7) in 214 deaths among a cohort of 728 patients with HAE identified from family history and corresponding family pedigrees.^14^ In addition, a positive diagnosis has been shown to improve quality of life and reduce disease burden for patients.^15^ Many gastroenterologists are likely to encounter patients with recurrent, unexplained abdominal pain^16^ and thus may be more likely to make the diagnosis of HAE compared with primary care physicians. We aimed to highlight the challenges of diagnosing HAE in patients who present with predominant GI symptoms and reviewed the existing body of literature.

## LITERATURE SEARCH METHODOLOGY

PubMed was searched for articles in English published from January 1, 2010, to July 31, 2020, using the following search terms: “hereditary angioedema” AND (“gastrointestinal” OR “abdominal” OR “Crohns” OR “irritable bowel syndrome” OR “bowel obstruction” OR “gastroenteritis”) AND (“diagnos*” OR “misdiagnos*”). Case studies were included, and conference abstracts were excluded. A total of 148 articles were identified and reviewed manually for relevance. Of these articles, 45 were determined to include relevant information on HAE and GI symptoms and were included in this review.

## SIGNS AND SYMPTOMS OF HAE

Although HAE symptoms generally begin in childhood,^8^ the average age at HAE diagnosis ranges from early childhood^17^ and early adulthood^18^ to the early 40s after a long diagnostic delay.^19^,^20^ Although HAE should occur in both male and female patients in similar proportions, owing to the autosomal dominance of the disease, it seems to be a female-predominant disease, which may reflect the symptom-exacerbating effects of estrogen.^21^,^22^ Estrogen can activate factor XII conversion to factor XIIa, which can activate the kallikrein pathway, leading to increased bradykinin production.^1^ Clinical studies and case reports have indicated that female patients are affected by HAE more severely and experience HAE attacks with greater frequency than male patients.^21^–^24^ As a female patient reaches puberty and experiences menstruation, ovulation, pregnancy, and menopause, the severity and frequency of attacks may change owing to normal fluctuations in estrogen.^25^,^26^ Menstruation, ovulation, estrogen-containing contraceptive use, and estrogen replacement in menopause have all been individually reported to trigger HAE attacks.^25^,^26^

Involvement of the GI tract with resultant abdominal pain occurs in 43% to 93% of patients with HAE, and up to 80% of all patients have recurrent abdominal pain.^27^ In a study of 149 patients with HAE who experienced 521 HAE attacks with any abdominal symptoms, 49% of the attacks were characterized by isolated abdominal pain.^28^ In an additional 33% of the attacks, patients had abdominal pain and symptoms at 1 other location outside of the abdomen.^28^ The most common abdominal symptoms were distension (77%), cramping (73%), and nausea (67%), whereas vomiting (21%) and diarrhea (14%) were much less common. However, other studies have reported much higher rates of vomiting (78%) and diarrhea (65%) in patients with abdominal symptoms of HAE.^29^

## DELAYS IN DIAGNOSIS OF HAE

Of 19 prospective and retrospective studies identified in this review, 9 reported delays in diagnosis experienced by patients with HAE with GI symptoms. The majority of studies were from Asia (4 studies) and Brazil (3 studies), with 1 study from the Czech Republic and 1 study from the United States.^17^–^20^,^30^–^34^ Because of geographic variation of disease and medical practices, it is unclear whether these data can be considered representative of HAE globally. The median age at onset of symptoms ranged from 5.7 years in the United States^17^ and 6.5 years in Brazil^18^ to 28 years in Korea.^31^ The proportion of patients reporting GI symptoms before diagnosis ranged from 18% in Taiwan^34^ to 86% in Brazil.^30^ Most studies reported a high proportion of patients with a family history of HAE or other angioedema (ranging from 59% to 100% of patients); despite this, delays in diagnosis were high, with average delays of 6 to 23 years (Table, Supplemental Digital Content 1, http://links.lww.com/JCG/A881).^17^–^20^,^30^–^34^

Observations from 25 cases reported since 2010 support these findings.^35^–^55^ One study reported apparent improvements in the time from symptom onset to diagnosis for patients with a more recent year of birth compared with older patients.^56^ However, we found no clear relationships between family history of HAE and age at diagnosis (Figure, Supplemental Digital Content 2, http://links.lww.com/JCG/A882).

Under-recognition and misdiagnoses in patients with HAE are key factors in diagnostic delays. In 418 patients with HAE, 44.3% (185/418) received 1 or more diagnoses before being diagnosed with HAE.^57^ The most common prior diagnoses were allergic angioedema 55.7% (103/185), appendicitis 27.0% (50/185), and other GI disorders including peptic ulcers and gastroesophageal reflux disease.^57^ Misdiagnosis rates were similar between female (46.5%) and male (41.1%) patients, and the types of prior diagnoses were similar, as well.^57^ A recent review of HAE focusing on GI manifestations concurred that HAE may present with nonspecific signs or symptoms resembling those of more common GI disorders.^16^

Delays in diagnosis of HAE were also found to increase the likelihood of unnecessary or exploratory surgeries or procedures and an increased risk of complications in patients presenting with GI symptoms.^32^,^56^,^58^ One study found that patients subsequently diagnosed with HAE were 2.5 times more likely to have had abdominal surgeries, 2.6 times more likely to have had appendectomies, and 2.3 times more likely to have had endoscopies than patients without HAE.^56^

## HEALTH CARE UTILIZATION

In the United States, stomach and abdominal pain is the chief complaint in 8% of emergency department (ED) visits.^59^ Patients diagnosed with HAE who experience severe abdominal pain as a presenting symptom are often first seen in an ED or by their primary care physician and may be referred to a gastroenterologist or surgeon.^60^,^61^ A US study conducted from 2006 to 2007 reported that there were 5040 ED visits for HAE, of which 2059 (40.9%) resulted in hospitalization.^62^ ED visits due to HAE were estimated to cost an average of $1479 (95% confidence interval, $1028 to $1929) per visit.^62^ A more recent study (2015 to 2016) reported the mean total annual cost of ED care was $32,939,152 for patients with HAE, with mean costs of $3598 per visit.^63^ Diagnosis and initiation of an appropriate treatment plan in patients with HAE can reduce the economic impact and burden of illness of HAE.

## DIAGNOSING HAE

A summary of diagnostic criteria extracted from published guidelines is provided in Table 1. Although many papers on the diagnosis and management of HAE have been published,^6^,^9^–^12^ at the time of this review existing guidelines (Table 1) typically assume that HAE is already included in the differential diagnosis. The International Consensus Algorithm for HAE, published in 2010, includes recurrent episodes of abdominal pain and vomiting as 1 of 4 primary criteria along with recurrent angioedema (without urticaria), laryngeal edema, and family history of angioedema.^11^ Brazilian guidelines for the diagnosis list abdominal pain of undefined organic etiology lasting longer than 6 hours as 1 of 4 primary criteria for clinical diagnosis of HAE.^9^ Noninflammatory subcutaneous angioedema lasting longer than 12 hours and recurrent laryngeal edema are additional primary clinical criteria. Family history is listed as a secondary clinical criterion. However, because up to 25% of patients with HAE can have de novo *SERPING1* mutations,^64^ a sizable proportion of patients with HAE will not have a family history of the disease. Lack of response to conventional doses of antihistamines is another indicator that a patient may have HAE as opposed to recurrent histaminergic angioedema, although this criterion has its limitations because patients with isolated angioedema may respond to other mast cell-targeted medications such as montelukast or omalizumab.

**TABLE 1:** Summary of Key Criteria for the Diagnosis of HAE for Published Guidance

Category	US HAEA Guidelines^8^	Brazilian Guidelines^9^	WAO/EAACI Guidelines^6^	International Consensus Guidelines for Pediatric Patients^10^	International Consensus Algorithm^11^	International/ Canadian HAE Guideline^12^
Primary criteria	Recurrent cutaneous angioedema (without urticaria)Abdominal symptomsOropharyngeal/laryngeal swelling	Noninflammatory subcutaneous angioedema lasting longer than 12 hAbdominal pain of undefined organic etiology lasting longer than 6 hRecurrent laryngeal edema	History of recurrent angioedema attacks	Pediatric patient with angioedema of unknown etiologyFamily history	Recurrent angioedema (without urticaria)Recurrent episodes of abdominal pain and vomitingLaryngeal edemaFamily history of angioedema	Recurrent angioedema (without urticaria)Recurrent abdominal pain/swelling
Secondary criteria	Screening of first-degree relatives	Family history of angioedema	Family history of HAEOnset of symptoms in childhood or adolescenceRecurrent and painful abdominal symptomsOccurrence of upper airway edemaFailure to respond to antihistamines, glucocorticoids, or epinephrinePresence of prodromal signs or symptoms before swellingsAbsence of urticaria			No response to allergy treatments
Laboratory tests	C4 levelC1-INH functional and antigenic level	Quantitative C1-INH, <50% in 2 distinct samplesFunctional C1-INH, <50% in 2 distinct samplesMutation in *SERPING1*	C4 levelC1-INH functional and antigenic level	C4 levelC1-INH functional and antigenic level	C4 levelC1-INH functional and antigenic level	C4 levelC1-INH functional and antigenic level

C1-INH indicates C1 inhibitor; EAACI, European Academy of Allergy and Clinical Immunology; HAE, hereditary angioedema; US HAEA, United States Hereditary Angioedema Association; WAO, World Allergy Organization.

International/Canadian guidelines on the management of HAE were published in 2019.^12^ Although not the focus of the guideline, the guidance relating to diagnosis notes that “HAE-C1-INH should be suspected in patients who have recurrent angioedema without concomitant urticaria and also in patients who have recurrent abdominal pain for which no cause is identified, particularly if there is a family history.” The authors encourage caution in patients who are prescribed angiotensin-converting enzyme inhibitors or estrogen-containing oral contraceptives, as although these agents may cause angioedema, their use does not rule out underlying HAE.

**FIGURE 2:**
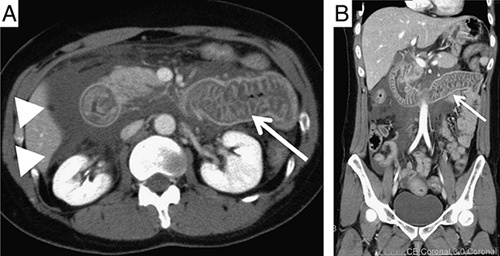
A and B, Abdominal computed tomography images during an attack of hereditary angioedema revealed intestinal edema (arrow) and ascites (arrowheads). Reproduced without changes under Creative Commons Attribution-NonCommercial 4.0 International license from Hirose et al.^60^

Insights from an international expert panel that considered opportunities for improvement in the patient journey of people with HAE were split into (1) onset of symptoms and initial evaluation, (2) referral and diagnosis, and (3) management of HAE.^65^ Key findings were that readily available information for patients and health care providers, including emergency physicians, is lacking and that health care providers may refer patients to physicians other than allergists or immunologists, resulting in difficulties in formulating global guidance. However, several tools to aid diagnosis of patients with HAE and GI symptoms have been published since 2010. In a review of the role of the health care provider in diagnosing HAE in patients presenting with unexplained abdominal pain, the authors note that unexplained abdominal pain, particularly when accompanied by swelling of the face and extremities, suggests the diagnosis of HAE.^16^ A family history of HAE and radiologic imaging (Fig. 2) demonstrating edematous bowel can also support the diagnosis. An alphabetic mnemonic can also be used in cases where HAE is suspected, where A=angioedema, B=bradykinin, C=C1-INH, D=distress factors, E=epinephrine nonresponsive, F=family history, and G=glottis/GI edema.^66^

## WHEN SHOULD HAE BE SUSPECTED? PRACTICAL GUIDANCE

Evidence of the HAE-GI symptom overlap comes from both observational^57^ and single-case studies^27^,^38^,^42^,^50^,^67^–^70^ (Table 2). HAE should be suspected when a patient presents with a history of recurrent cutaneous angioedema attacks.^3^ Secondary considerations include family history of HAE; onset of symptoms in childhood/adolescence; recurrent and painful abdominal symptoms; occurrence of upper airway edema; swelling that fails to respond to antihistamines, glucocorticoids, or epinephrine; presence of prodromal signs or symptoms before attacks; and the absence of urticaria.^3^ Once HAE is suspected, laboratory investigations (ie, C4, C1-INH level and function) should be carried out regardless of absence or presence of painful abdominal symptoms, as C1-INH levels (Type 1) and C1-INH function (Type 1 and 2) are consistently low during both asymptomatic and symptomatic periods. C4 levels are consistently low in most (>85%) individuals with HAE and will be low in all patients during an angioedema attack. For a diagnosis of HAE, a history of recurrent cutaneous angioedema attacks, and a low (<50% of normal) C1-INH result are required; a low C4 (at baseline or during attack) is also expected.^8^

**TABLE 2:** Gastrointestinal Disorders That Overlap the Abdominal Symptoms of HAE

Celiac disease	Small bowel obstruction
Crohn’s disease	Familial Mediterranean fever
Ulcerative colitis	Celiac artery compression/median arcuate ligament syndrome
Irritable bowel syndrome	Mast cell activation syndrome
Functional dyspepsia	Mast cell enterocolitis
Chronic nausea and vomiting	Migraines with an abdominal component
Cyclic vomiting syndrome	Porphyrias, including acute intermittent porphyria

HAE indicates hereditary angioedema.

Episodic abdominal pain; recurrent small bowel obstruction without a surgical history and no clear etiology; or nausea, vomiting, and/or diarrhea with complete resolution between episodes warrant consideration of HAE as part of the differential diagnosis. Pertinent history may include recurrent episodes of cutaneous angioedema without pruritus and urticaria.^70^ Factors to consider in recognizing HAE in patients with GI symptoms are summarized in Table 3. Although most HAE attacks have no identifiable trigger, in females, HAE attacks may be brought on by normal hormonal fluctuations or the use of medications such as estrogen-containing oral contraceptives.^3^ In HAE, recurrent abdominal pain may vary in severity but typically occurs as severe intermittent episodes lasting 2 to 5 days if untreated, with normal asymptomatic periods between attacks.^27^ Abdominal pain in HAE is often described as cramping, colicky, tiring, or exhausting.^27^,^28^,^71^ During an attack, patients may have abdominal distension, tenderness, and possibly ascites.^72^ Ascites was detected in 92% of HAE patients who underwent abdominal ultrasound during an abdominal HAE attack.^73^ In our clinical experience, fever, peritoneal signs, or elevated white blood cell counts are not common during HAE attacks. These signs and symptoms would be more common with an acute abdominal pathology or familial Mediterranean fever.^70^ Elevated neutrophil counts (without increased bands), hypovolemia from fluid losses, and hemoconcentration have been reported in severe attacks.^19^ Nonresponse to glucocorticoids, antihistamines, or epinephrine is an additional indication that a mechanism other than histamine-mediated angioedema is involved.^9^,^11^,^12^

**TABLE 3:** Factors for Consideration in Recognizing Patients With HAE

Parameter	Key Factors
Patient history	Recurrent unexplained gastrointestinal painUse of estrogen-containing contraceptivesPatient has recently reached puberty, is pregnant, or has recently entered menopausePrevious abdominal diagnoses and/or surgical interventions
Family history	Family history of unexplained swelling or HAE
Signs and symptoms at presentation	No fever, peritoneal signs, or elevated white blood cell countSevere abdominal pain without a history of laryngeal or cutaneous swelling
Clinical assessments	Test for low serum level of C4 and low C1-INH level or function*Use CT or ultrasonography of the abdomen or pelvis
Action	Refer patients to a physician experienced in managing HAE for confirmation of diagnosis

* Both C1-INH antigenic level and functional level should be tested to distinguish between HAE type I (low antigen and low function) and HAE type II (normal antigen and low function).

C1-INH indicates C1 inhibitor; CT, contrast-enhanced computed tomography; HAE, hereditary angioedema.

There are limited data on the prevalence of HAE among the various causes of chronic abdominal pain, but many patients will have episodes with isolated abdominal pain and angioedema in the absence of any skin or airway swelling. Over the course of a lifetime, however, it is very unusual to solely experience abdominal symptoms. Most patients will have recurrent cutaneous angioedema as well, but this may not occur with every episode. Thus, connecting recurrent skin symptoms with the recurrent abdominal pain (which may be occurring at different times) is a key clinical clue.

In the diagnostic evaluation, taking a comprehensive family history and asking about a personal history of cutaneous swelling or recurrent airway symptoms, along with clinical suspicion of HAE, can aid in differentiating HAE from other causes of abdominal pain. Health care providers should consider HAE in patients with severe abdominal pain even in the absence of a history of laryngeal or cutaneous swelling. Contrast-enhanced computed tomography or ultrasonography of the abdomen and pelvis during a symptomatic episode is recommended to evaluate for GI angioedema and transitory ascites if the cause of symptoms is unclear.^74^–^76^ Imaging findings in a patient with an HAE attack may include intestinal wall edema^76^ early in an attack, whereas isolated ascites may be seen later in the course of an attack. Abdominal contrast-enhanced computed tomography may also show prominent mesenteric vessels during HAE attacks. However, the sensitivity of abdominal imaging in detecting abnormalities during HAE abdominal symptoms is unknown. Upon reimaging after the abdominal pain has resolved, complete remission of abnormal findings may support the diagnosis of HAE.^76^ Determining serum levels of C4 constitutes the principal screening test for HAE.^8^,^9^,^11^ C4 levels are persistently low in most individuals with HAE due to C1-INH deficiency, although a small subset of patients demonstrates only low C4 levels during a symptomatic episode. If C4 levels are low at baseline or during an attack, then testing for levels and activity of C1-INH is indicated.^8^,^9^,^11^,^12^ Patients who are suspected of having HAE by initial laboratory screen or imaging should be referred to an allergist, immunologist, or other physician familiar with managing HAE for confirmation of diagnosis.^72^

## A BRIEF WORD ON HAE WITH NORMAL C1-INH

Patients with HAE with normal C1-INH (HAE-nl-C1-INH) have also been described;^77^,^78^ however, the pathology is not as well understood as that of HAE-C1-INH. The causes of HAE-nl-C1-INH have been ascribed to underlying genetic mutations in the genes for factor XII,^79^ plasminogen,^80^ angiopoietin-1,^81^ kininogen 1,^82^ myoferlin,^83^ and heparan sulfate-glucosamine 3-O-sulfotransferase 6.^84^ In an additional subset of patients,^85^ the responsible mutation has not been defined. The clinical presentation of HAE-nl-C1-INH is similar to that of HAE-C1-INH but with some differences, including more frequent swelling of the face and tongue rather than abdominal symptoms.^85^ Because the underlying causes of HAE-nl-C1-INH are genetic mutations, genetic testing may be helpful in the diagnosis of these patients. However, due to the nuances and difficulty in establishing a diagnosis of HAE-nl-C1-INH, and because confirmatory biomarkers are not readily available,^86^ management of these patients should be discussed with an HAE specialist before committing to treatment.^8^ For the purposes of this review, we have focused on patients with HAE-C1-INH because they account for the vast majority of patients with HAE.

## CURRENTLY RECOMMENDED TREATMENTS FOR HAE

The international guidelines developed by the World Allergy Organization and the European Academy of Allergy and Clinical Immunology were recently updated to provide a global standard for HAE management.^87^ These are similar to the updated guidelines by the US HAE Association Medical Advisory Board.^8^ Treatment recommendations comprise on-demand treatment of HAE attacks, short-term prophylaxis, and long-term prophylaxis. The preferred on-demand treatments for HAE attacks are icatibant (Firazyr, Takeda), ecallantide (Kalbitor, Takeda), and intravenous (IV) plasma-derived C1-INH (Berinert, CSL Behring; Cinryze, Takeda – FDA-approved for long-term prophylaxis only) or IV recombinant human C1-INH (Ruconest, Pharming). For short-term prophylaxis, it is recommended to use IV plasma-derived C1-INH. The preferred long-term prophylactic treatments are plasma-derived C1-INH administered SC (Haegarda, CSL Behring) or IV (Cinryze, Takeda), lanadelumab (Takhzyro, Takeda), and berotralstat (Orladeyo, Biocryst).

## CONCLUSION

Patients with undiagnosed HAE with GI symptoms are likely to present across many care settings, including both primary care and specialist clinics. Inclusion of HAE as part of the differential diagnosis in patients with unexplained severe and often recurrent abdominal pain, with or without skin manifestations, may improve the diagnosis rate of HAE. Careful history-taking and a physical examination along with selected imaging studies and laboratory testing are vital to making the diagnosis. By including HAE in the differential diagnosis, providers can make the appropriate referral to a physician experienced in managing HAE, thus shortening the time to diagnosis and effective treatment and reducing the burden of disease.

## Supplementary Material

SUPPLEMENTARY MATERIAL

Supplemental Digital Content is available for this article. Direct URL citations appear in the printed text and are provided in the HTML and PDF versions of this article on the journal's website, www.jcge.com.
