# Supplementary material for: Consider Hereditary Angioedema in the Differential Diagnosis for Unexplained Recurring Abdominal Pain
Source: J Clin Gastroenterol. 2022 Aug 15;56(9):740–7. doi: 10.1097/MCG.0000000000001744 (PMC9432812; doi:10.1097/MCG.0000000000001744)
Supplement: SUPPLEMENTARY MATERIAL [file mcg-56-740-s001.docx]

Supplemental Materials

TABLE, SUPPLEMENTAL DIGITAL CONTENT 1. Delay in Diagnosis of HAE in Prospective and Retrospective Studies

| Reference | Type of Study | N | Female Patients,  n (%) | Patients with GI Symptoms Prior to HAE Diagnosis, % | Age at Symptom Onset, Years | Age at Diagnosis, Years | Delay in Diagnosis, Years | Family History of HAE, % |
| --- | --- | --- | --- | --- | --- | --- | --- | --- |
| Alonso 2020^29^ | Prospective | 107 | 72 (67.3) | 89 | Mean (SD) 11.8 (9.6) | Mean (SD) 29.0 (14.6) | Mean (range) 17.7 (0-61) | 86.9 |
| Grumach 2013^17^ | Registry | 210 | 133 (63) | 54 | Median 6.5 | Median 21 | Median 14.5 | 78.1 |
| Ohsawa 2014^19^ | Retrospective | 72 | 13 (18.1) | 60.8 | Mean (SD) 19.5 (8.0) | Mean (SD) 42.3 (14.3) [first visit] | Mean 22.8 | 82.6 |
| Jung 2018^30^ | Retrospective | 65 | 44 (67.7) | 40.5 | Mean (SD) 28.4 (14.1) | Mean (SD) 36.5 (15.8) | Mean (SD) 7.8 (10.5) | 58.7 |
| Hakl 2016^32^ | Retrospective | 77 | 43 (55.8) | 40.6 | Median (range) 17  (1-40) | – | Median (range)  7 (0-42) | 33.8 |
| Fragnan 2018^31^ | Retrospective | 51 | 38 (74.5) | 84.8 | Median (range) 11 (22-41) | – | Median (range) 13 (0.3-50) | 70.6 |
| Nanda 2015^16^ | Retrospective | 21 | (6) | 93 | Median (range) 5.7 (5-9) | Median (range) 5.0 (4-8) | Medians 0.9^a^ and 6^b^ | 86 |
| Lei 2011^33^ | Retrospective | 19 | 8 (42.1) | 18 | Mean (SD) 20.81 (7.88) | Mean (SD) 29.27 (16.47) [symptomatic] | Mean (SD) 8.5 (11.0) | 100^c^ |
| Ohsawa 2013^18^ | Retrospective | 14 | 9 (64.3) | 43 | Mean (SD) 20.2 (9.4) | – | Mean (SD) 22.7 (14.2) | 86 |

^a^Patients with a family history.

^b^Patients without a family history.

^c^Based on study collection criteria.

GI indicates gastrointestinal; HAE, hereditary angioedema; SD, standard deviation.
