# Supplementary material for: Consider Hereditary Angioedema in the Differential Diagnosis for Unexplained Recurring Abdominal Pain
Source: J Clin Gastroenterol. 2022 Aug 15;56(9):740–7. doi: 10.1097/MCG.0000000000001744 (PMC9432812; doi:10.1097/MCG.0000000000001744)
Supplement: SUPPLEMENTARY MATERIAL [file mcg-56-740-s002.pdf]

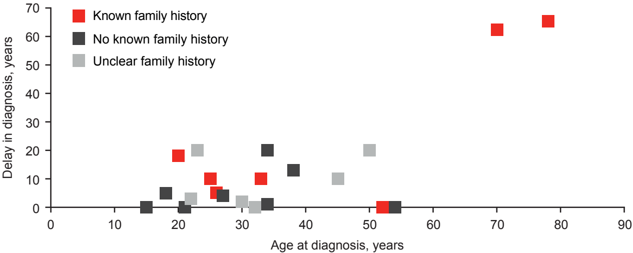

FIGURE, SUPPLEMENTAL DIGITAL CONTENT 2. Case studies of patients with gastrointestinal symptoms prior to diagnosis of hereditary angioedema show there is no relationship between family history and age at diagnosis.[34-55]
